# Longitudinal Stability of the Gut Microbiota in Relapsing–Remitting Multiple Sclerosis: A Prospective Observational Study

**DOI:** 10.3390/microorganisms14092080

**Published:** 2026-09-17

**Authors:** Cristiana Vacaras, Vitalie Vacaras, Cristina Nistor, Cristina Jucan, Ioana Monica Furdui, Stefan Cristian Vesa, Stefan Strilciuc, Emanuel Ștefănescu, Diana Chira, Alexandru Rafila, Dafin F. Muresanu

**Affiliations:** 1 Neurology Department, Cluj Emergency County Hospital, 400012 Cluj-Napoca, Romania; 2 Department of Neurosciences, Faculty of Medicine, Iuliu Hațieganu University of Medicine and Pharmacy, 400012 Cluj-Napoca, Romania; 3 Department of Pharmacology, Toxicology and Clinical Pharmacology, Faculty of Medicine, Iuliu Haţieganu University of Medicine and Pharmacy, 400349 Cluj-Napoca, Romania; 4 Department of Genomics, MEDFUTURE Institute for Biomedical Research, Iuliu Hațieganu University of Medicine and Pharmacy, 400337 Cluj-Napoca, Romania; 5 RoNeuro Institute for Neurological Research and Diagnostic, 400364 Cluj-Napoca, Romania; 6 Department of Microbiology, Carol Davila University of Medicine and Pharmacy, Dionisie Lupu Street, 050474 Bucharest, Romania

**Keywords:** multiple sclerosis, gastrointestinal microbiome, metagenomics, biomarker, disease progression

## Abstract

The gut microbiota plays a key role in immune regulation in multiple sclerosis (MS), yet longitudinal data on microbiome dynamics and their relationships with disability and biomarkers remain limited. This study aimed to identify longitudinal gut microbiome changes in patients with relapsing-remitting multiple sclerosis (RRMS) and their association with disability and specific serum biomarkers, including predicted metabolic pathways. In this prospective longitudinal study, 29 treatment-naïve RRMS patients were evaluated at baseline and after one year of disease-modifying therapy. Clinical assessments included the Expanded Disability Status Scale (EDSS) and disability tests. Serum glial fibrillary acidic protein (GFAP), neurofilament light chain (NfL), and S100B protein concentrations were measured at both visits. Gut microbiota composition was characterized by shotgun metagenomic sequencing, including taxonomic composition, diversity measures, functional pathways, gut metabolic modules, and gut–brain modules. In the 29 patients, overall gut microbiota composition and diversity remained stable during follow-up. Although EDSS progression was associated with a trend toward greater microbiome instability, this association did not reach statistical significance. Several bacterial taxa and predicted metabolic pathways showed nominal associations with disability, but were not significant after correction. GFAP levels increased significantly over time, whereas NfL levels decreased and S100B levels remained unchanged. Early RRMS is characterized by a stable gut microbiome during the first year of treatment. Exploratory taxonomic and functional associations did not remain significant after correction for multiple testing, highlighting the need for larger longitudinal cohorts.

## 1. Introduction

Multiple sclerosis (MS) is an immune-mediated disease of the central nervous system with a complex etiology and heterogeneous clinical manifestations [1]. Its pathogenesis involves the activation of autoreactive T-cells, which cross the blood–brain barrier (BBB) and initiate inflammatory cascades and axonal degeneration, ultimately leading to demyelinating lesions. MS commonly affects young adults, particularly women, and affects more than 2.3 million people worldwide, resulting in substantial neurological disability and a major impact on quality of life [2]. The majority of patients present with the RRMS form, characterized by clinical relapses of new or worsening neurological symptoms, followed by periods without evident disease progression [2].

The gut microbiota has emerged as an important modulator of immune function in various immune-mediated diseases, including MS [1]. Gut microorganisms from the human gastrointestinal tract contribute to host physiology through nutrient metabolism, maintenance of intestinal barrier integrity, immune regulation, and the production of bioactive metabolites [2,3]. Alterations in gut microbial composition have been reported in people with MS compared with healthy controls, although the taxonomic findings vary considerably across studies and no consistent MS-specific microbial signature has been established [4].

The gut–brain axis encompasses bidirectional communication between the gastrointestinal tract and the central nervous system through immune, endocrine, metabolic, and neural pathways [5]. Among microbiota-derived metabolites, short-chain fatty acids (SCFAs)—primarily acetate, propionate, and butyrate—are generated through bacterial fermentation of dietary carbohydrates and have been implicated in immune and gut–brain signaling [6]. Experimental and clinical evidence suggests that SCFAs can influence regulatory and effector T-cell responses and may modulate blood–brain barrier integrity and neuroimmune function [6]. In MS specifically, reduced circulating and fecal propionate concentrations have been reported, while propionate supplementation has been associated with increased regulatory T-cell frequency and reduced Th1/Th17 cells, supporting a potential immunomodulatory role for microbial SCFA metabolism in MS [7].

A healthy microbiota is characterized by stability, defined as the capacity to maintain a relatively unchanged state, and resilience, defined as the ability to return to its original state following perturbation [8]. An altered microbial structure and impaired resilience lead to dysbiosis [9].

Serum biomarkers such as Glial Fibrillary Acidic Protein (GFAP) and Neurofilament Light Chain (NfL) play important roles in MS [10]. NfL is a well-established marker of inflammation and neuroaxonal injury, with higher levels observed in PwMS compared with healthy controls, although it does not differentiate between MS types. It is used as a prognostic marker, as higher levels are associated with increased relapse risk and the development of new or active MRI lesions. GFAP reflects astrocytic damage and is increased in progressive MS compared with RRMS. GFAP levels appear to correlate with disease progression and disability, including EDSS score and cognitive decline [10,11]. Regarding S100B, fewer studies have investigated its relationship with MS. It is considered an exploratory biomarker of glial and neuronal injury and a mediator of inflammatory responses [12].

Although previous longitudinal studies have investigated gut microbiome dynamics in MS, evidence remains limited, and relatively few studies have integrated longitudinal microbiome profiling with concurrent measures of neurological disability and circulating markers of neuroaxonal and astroglial injury. In addition, functional characterization of the microbiome over time remains insufficiently explored. The present study addresses these gaps by longitudinally evaluating treatment-naïve patients with RRMS from baseline through one year after DMT initiation, using shotgun metagenomic sequencing to characterize both taxonomic composition and predicted functional potential, together with standardized disability assessments and serial measurements of serum NfL, GFAP, and S100B. This integrated longitudinal approach enables the simultaneous assessment of within-patient microbiome dynamics and their relationship with clinical and biological changes during early treated RRMS.

We hypothesized that within-person changes in microbial composition and predicted functional potential over one year would be accompanied by and correlate with changes in disability and serum neurological biomarkers.

The aims of this study were to identify longitudinal changes in gut microbiome composition and predicted microbial functional potential in treatment-naïve patients with RRMS, to analyze the relationship between microbiome dynamics, disability progression, clinical outcomes, and serum biomarkers, and to explore potential metabolic pathways involved in the gut–brain axis.

## 2. Materials and Methods

### 2.1. Subjects

This prospective, observational, longitudinal study included 29 treatment-naïve adult patients with RRMS diagnosed according to the revised 2017 McDonald diagnostic criteria [13]. Patients were recruited from the Neurology Department of the Cluj-Napoca County Emergency Hospital, Romania, between February 2024 and August 2025. Ethical approval was obtained, and all participants provided written informed consent.

Patients were excluded if they had inflammatory or gastrointestinal disorders (hepatitis, gastritis, Crohn’s disease, ulcerative colitis, diverticulitis, cholecystitis), were pregnant or breastfeeding, or had received, in the preceding 3 months, treatment with probiotics, antivirals, antibiotics, non-steroidal anti-inflammatory drugs, corticosteroids, or proton-pump inhibitors.

### 2.2. Sample Collection

Subjects were evaluated at two follow-up visits: at study onset (V0), before initiation of any disease-modifying treatment (DMT), and approximately one year after starting DMT (V1). At each visit, clinical data, EDSS score, demographic and disease characteristic questionnaires, fecal samples, and blood samples were collected. Standardized tests assessing MS disability included the timed 25-foot walk test (T25FW), 9-hole peg test (9HPT), and symbol digit modalities test (SDMT).

The EDSS score evaluates neurological disability in MS, based on several functional systems [14]. In our study, EDSS scores were categorized into two groups: mild disability (≤2.5 points) and moderate/severe disability (>2.5 points). EDSS progression was defined as an increase of >0.5 points. T25FW is a gait evaluation method that measures the time (in seconds) required for an individual to walk 25 feet as quickly as possible [15]. 9HPT quantifies upper extremity coordination using a standardized device, for both dominant (9HPT-D) and non-dominant (9HPT-ND) hands [16]. The written Symbol Digit Modalities Test (SDMT) is a standardized cognitive assessment evaluating information processing speed and sustained attention, and it can reflect both cognitive and upper extremity motor function [17].

The Rapid Eating Assessment for Participants (REAP) is a brief questionnaire used to assess dietary habits in clinical and research settings [18]. We used the short version of REAP, a 16-item self-administered questionnaire designed for the rapid, semi-quantitative evaluation of diet quality. It covers the main food groups and eating behaviours. The first 13 REAP items were summed to generate a total score ranging from 13 to 39, with higher scores indicating healthier dietary habits. Scores were classified as excellent (35–39), good (30–34), satisfactory (25–29), unsatisfactory (20–24), or very unhealthy (13–19) dietary habits.

Peripheral blood samples were collected at both study visits and processed for serum separation. Blood was centrifuged, after which the serum was aliquoted and stored at −20 °C until analysis. Serum biomarker concentrations were quantified using commercially available ELISA kits according to the manufacturers’ instructions. The analytical measurement ranges were 15.63–1000 pg/mL for GFAP (Human GFAP ELISA Kit, EEL079), 1.6–1200 pg/mL for NfL (Human NF-L ELISA Kit, BMS2362), and 31.25–2000 pg/mL for S100B (Human S100B ELISA Kit, EEL045), with analytical sensitivities of 9.38, 0.8, and 18.75 pg/mL, respectively. These analytical ranges represent assay-specific measurement intervals rather than clinical reference intervals.

Fecal samples were collected from each participant at baseline (V0) and at the one-year follow-up visit (V1). According to the manufacturer’s instructions, each sample was collected at any time of the day, using specific collection kits used for this type of analysis, stored at a constant temperature under 25 °C and subsequently shipped in January 2026 to the specialized laboratory for metagenomic analysis. The storage duration varied from a few days to two years, but it was accordingly to laboratory instructions, as fecal samples were stored in specialized preserving buffers and they were not subjected to any freeze–thaw cycles prior to DNA extraction.

The patients received DMT after enrolling in this study, selected according to disease type and other individual factors. Treatments were categorized into two groups: high-efficacy (HE) (Ocrelizumab, Ofatumumab, Natalizumab, Cladribine) and moderate-efficacy (ME) (teriflunomide, interferon, dimethyl fumarate). Clinical disease activity was assessed using the annualized relapse rate (ARR), calculated as the total number of relapses divided by the number of years since disease onset. During the one-year follow-up period, none of the participants experienced a clinical relapse or received corticosteroid treatment.

### 2.3. Shotgun Sequencing and Statistical Analysis

Shotgun sequencing is a metagenomic approach in which the DNA found in a biological sample is fragmented and sequenced without prior amplification of specific genetic markers. Unlike 16S rRNA gene sequencing, which targets only bacterial taxonomic markers, the shotgun method captures a complete taxonomic profile of microorganisms, including bacteria, archaea, viruses, and fungi. In addition, it provides information on the functional potential of the gut microbiome by identifying metabolic and signaling pathways involved in gut–brain communication [19].

Microbial DNA was extracted using the QIAGEN DNeasy PowerSoil Pro Kit according to the manufacturer’s protocol. Extracted DNA was quantified using a Qubit Flex fluorometer with the Qubit™ dsDNA 1× HS Assay Kit (Thermo Fisher Scientific, Waltham, MA, USA). DNA libraries were prepared using the Watchmaker DNA Library Prep Kit (7K0019-1K). Genomic DNA was fragmented using Watchmaker Frag/AT Buffer and Frag/AT Enzyme Mix, followed by addition of IDT xGen UDI primers and IDT Stubby Adapters and seven cycles of PCR amplification. Libraries were purified using CleanNGS magnetic beads (CleanNA, Waddinxveen, The Netherlands), eluted in nuclease-free water, and quantified using the Qubit™ dsDNA HS Assay Kit. Following circularization, libraries were sequenced on the Element AVITI platform using 2 × 150 bp paired-end sequencing.

Raw sequencing data were processed using the CosmosID-HUB bioinformatics pipeline. Human-derived reads were removed by mapping against the GRCh38.p14 human reference genome using Bowtie2 (v2.4.2). Adapter sequences and bases with Phred quality scores < 30 were removed using AdapterRemoval (v2.3.1), and only high-quality non-host paired reads ≥100 bp were retained for downstream analysis. These reads were mapped to the Clinical Microbiomics Human Microbiome Reference HMR05 gene catalog using BWA-MEM (v0.7.17). Species abundances were subsequently calculated from species-specific signature genes and normalized within each sample to generate relative abundance profiles.

The results included relative abundances of microbial taxa and functional potential profiles. Taxonomic analyses were performed at the species level, and relative abundances were estimated from species-specific signature genes using the HMR05 reference gene catalog and normalized within each sample to sum to 100%. Species detection and abundance estimation followed the quality and detection criteria implemented in the CosmosID-HUB pipeline. No additional study-specific abundance threshold was applied beyond the detection criteria implemented in the CosmosID-HUB pipeline.

Functional potential profiles, including Gut–Brain Modules (GBMs) and Gut Metabolic Modules (GMMs), were derived from the resulting species profiles, with each module defined as a series of enzymatic steps represented by Kyoto Encyclopedia of Genes and Genomes orthology (KO) identifiers [20]. GMMs and GBMs were used to characterize the predicted functional potential of the gut microbiome based on metagenomic species profiles. These module-based profiles reflect the genomic potential of the microbial community to perform specific metabolic functions and should not be interpreted as direct measurements of pathway activity or metabolite production.

Accordingly, SCFA- and tryptophan-related modules were interpreted as predicted microbial functional potential for the corresponding metabolic pathways rather than as direct measures of metabolic activity, metabolite production, or metabolite concentration. Neither SCFAs nor tryptophan-derived metabolites were directly quantified in this study.

Alpha diversity was assessed using the Chao, Shannon, and Simpson indices. Higher values indicate greater species richness and/or evenness [21]. Beta diversity, reflecting differences in microbial composition between samples, was evaluated using the Bray–Curtis and Jaccard indices. Longitudinal differences in beta diversity were assessed using PERMANOVA based on Bray–Curtis dissimilarity with 999 permutations, restricted within participant to account for the paired V0–V1 study design. Additional analyses included principal coordinate analysis (PCoA) and Linear Discriminant Analysis Effect Size (LEfSe), using an alpha value of 0.05 and a logarithmic LDA score threshold of 2.0. Paired comparisons between V0 and V1 were performed using the Wilcoxon signed-rank test. To examine whether longitudinal outcomes differed according to DMT efficacy category, within-participant changes from V0 to V1 in clinical measures, REAP score, serum biomarkers, and alpha-diversity indices were compared between the HE and ME groups using the Wilcoxon rank-sum test. Within-participant Bray–Curtis dissimilarity between V0 and V1 was compared using the same approach. Benjamini–Hochberg false discovery rate correction was applied across the 13 exploratory treatment-group comparisons. Associations between continuous or ordinal variables were assessed using Spearman rank correlation. For the exploratory microbiome association analyses, Benjamini–Hochberg false discovery rate (FDR) correction was applied separately within defined analytical families of tests. One family comprised the 301 taxon-level correlations with clinical, dietary, and age variables, whereas a second family comprised the 86 taxon-level correlations with disease duration and annualized relapse rate. FDR correction was also applied separately to the 15 selected taxon–serum biomarker associations, 15 longitudinal selected taxon–EDSS associations, 103 longitudinal GMM comparisons, 49 longitudinal GBM comparisons, 15 SCFA-related pathway associations, and six tryptophan-related pathway associations. In exploratory treatment-group analyses, FDR correction was applied separately within each family of tests: one family comprised the 13 comparisons involving clinical measures, REAP score, serum biomarkers, alpha-diversity indices, and Bray–Curtis dissimilarity, whereas the second family comprised the five selected LEfSe-derived taxa.

## 3. Results

### 3.1. Baseline Cohort Characteristics and Microbiome Composition

The median age of our cohort (n = 29) was 31 years; the majority were female (62.1%), with a median disease duration of 17 months, a median ARR of 1, and a median EDSS score of 1.5 points (Table 1).

From a taxonomic perspective, the baseline dominant phyla were Bacillota_A and Bacteroidota, with mean relative abundances of 55% and 33.3%, respectively, which together represented the majority of the microbial community. At the genus level, Prevotella, Faecalibacterium, Phocaeicola and Bacteroides were among the most abundant taxa (Supplementary Tables S1 and S2, Supplementary Figure S1).

### 3.2. Longitudinal Changes in Clinical Outcomes and Serum Biomarkers

**Figure 1:**
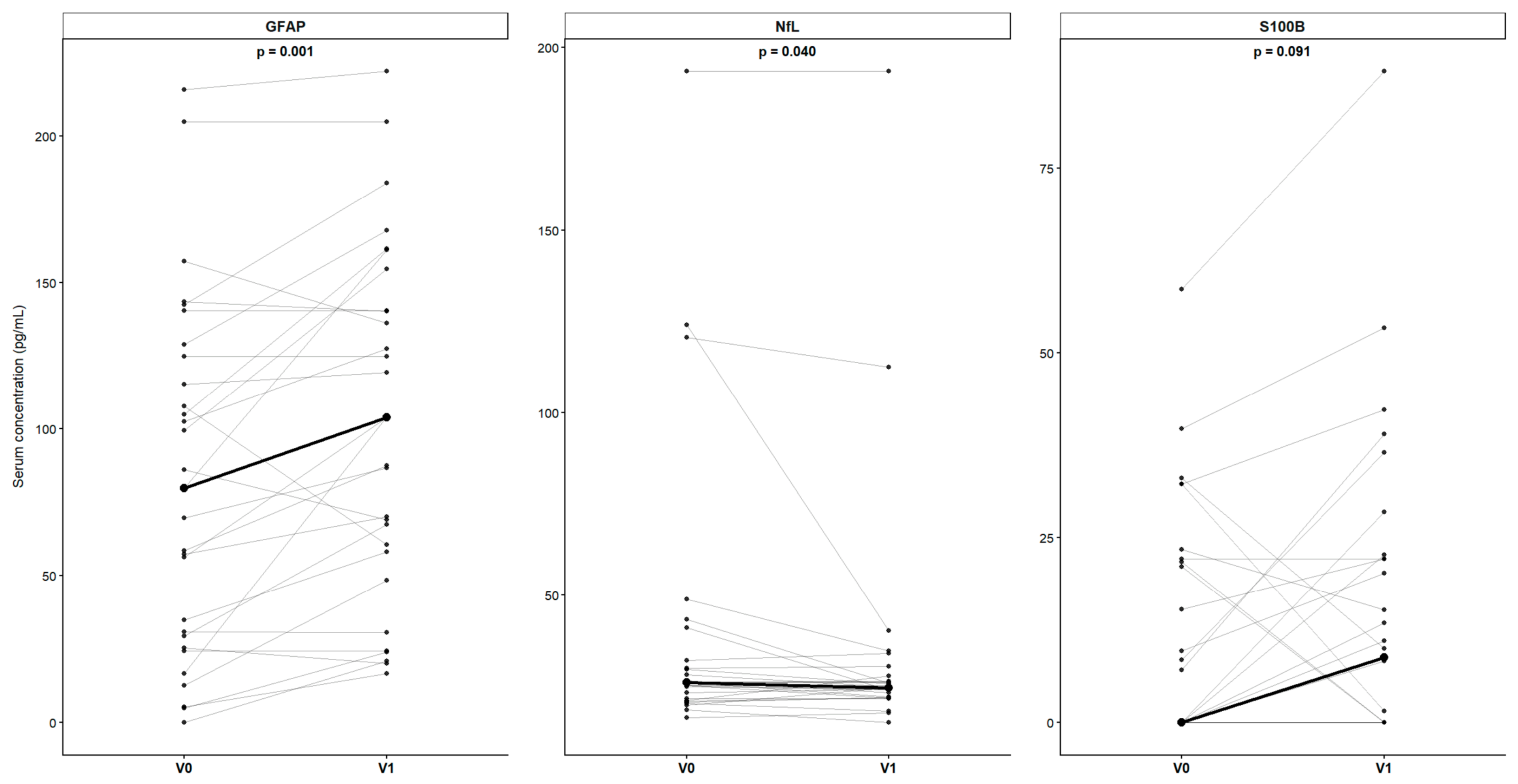
Individual longitudinal trajectories of serum neurological biomarkers between V0 and (V1). Individual paired results for each patient were joined by thin lines, while the thick line joined the median at each visit. GFAP concentrations increased significantly over time (*p* = 0.001), whereas NfL concentrations decreased modestly (*p* = 0.040). S100B concentrations increased numerically, but the change was not statistically significant (*p* = 0.091). The Wilcoxon signed-rank test was used to calculate *p*-values.

The analysis of serum biomarkers showed a significant increase in GFAP concentrations between baseline and follow-up (*p* = 0.001). In contrast, NfL levels significantly decreased between visits (*p* = 0.040), although the magnitude of this change was modest (Figure 1). S100B concentrations showed a non-significant trend toward higher levels at follow-up (*p* = 0.091).

Regarding categorical variables, no major changes were observed between visits. EDSS severity categories remained unchanged at both evaluations, with 23 (79.3%) patients classified as having mild disability and 6 (20.7%) as having moderate/severe disability (*p* = 1.000). Slight changes were observed in REAP dietary habit categories over time; however, most patients remained within the satisfactory category, and the overall distribution did not change significantly (*p* = 0.695). All results are detailed in Table 2.

### 3.3. Longitudinal Gut Microbiome Stability

At the taxonomic level, the gut microbiota showed a broadly similar composition between baseline and follow-up. No significant changes were observed in alpha diversity between baseline and follow-up, with Shannon diversity, Simpson diversity, and Chao1 richness remaining stable throughout the study period (Table 3, Supplementary Figure S2).

**Figure 2:**
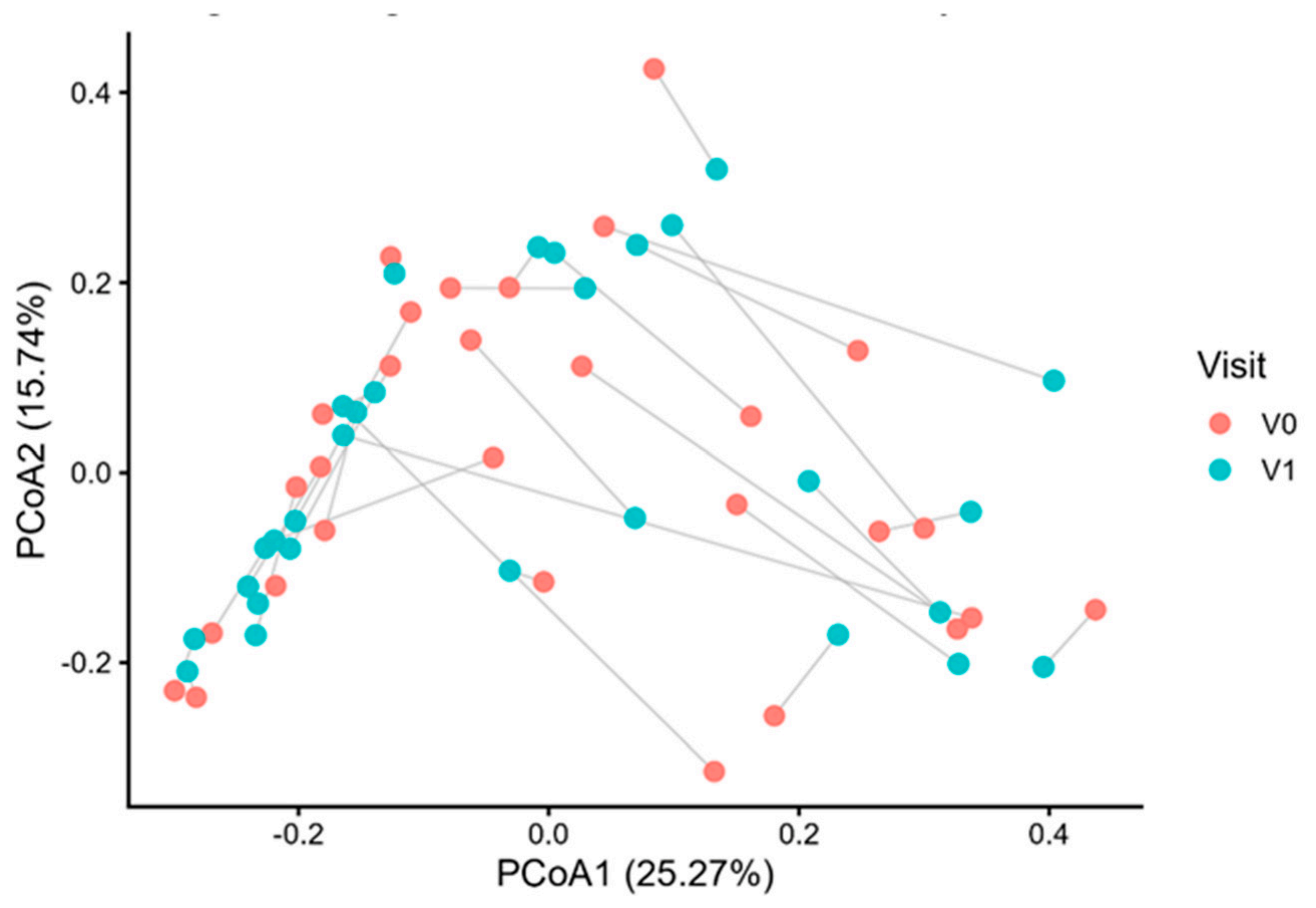
Bray–Curtis Principal coordinate analysis (PCoA), beta diversity between V1 vs. V0. Figure 2 represents a paired Principal Coordinates Analysis (PCoA) based on Bray–Curtis dissimilarity, illustrating the gut microbiome composition at baseline (V0) and the 1-year follow-up (V1). Each line connects paired samples from the same participant, highlighting individual longitudinal trajectories.

Beta-diversity analysis of our cohort revealed no significant global changes in microbial community composition after one year (PERMANOVA, Bray–Curtis dissimilarity: R^2^ = 0.008, F = 0.446, *p* = 0.436). However, individual Bray–Curtis dissimilarity values indicated a moderate within-patient microbiome variation over time (0.379 (0.314–0.448)). In the PCoA, the first two principal coordinates explained 25.27% and 15.74% of the total variance, respectively (Figure 2).

### 3.4. Exploratory Analysis According to DMT Efficacy Category

Exploratory analyses based on DMT efficacy class did not reveal significant differences between the HE group (n = 20) and the ME group (n = 9) in terms of changes over time in EDSS, other clinical assessments, diet score, serum biomarkers, or alpha-diversity indices. Individual Bray–Curtis dissimilarity also did not differ significantly between the HE and ME groups: median [IQR] 0.363 [0.294–0.438] and 0.479 [0.353–0.502], respectively; *p* = 0.114. None of the 13 comparisons was statistically significant, including after Benjamini–Hochberg FDR correction (all adjusted *p* ≥ 0.749) (Supplementary Table S3). Longitudinal changes in the five selected LEfSe-derived taxa also did not differ significantly between the HE and ME groups (all *p* ≥ 0.518; all FDR-adjusted *p* = 0.789).

### 3.5. Exploratory Associations with Disability

Individual Bray–Curtis dissimilarity showed a non-significant positive correlation with change in EDSS (rho = 0.321, *p* = 0.090), Supplementary Table S4.

Patients with EDSS progression (>0.5-point increase) exhibited greater microbiome instability than patients with stable or improved disability status (median Bray–Curtis dissimilarity: 0.439 vs. 0.369; mean: 0.443 vs. 0.373), although this difference did not reach statistical significance (*p* = 0.328).

Among LEfSe-derived candidate taxa, the five taxa with the strongest nominal associations with baseline EDSS were selected for longitudinal correlation analyses with EDSS at follow-up and changes in EDSS over time. These analyses showed directionally consistent nominal associations between these taxa and disability status across both study visits. *Spyradocola merdavium* showed consistent positive correlations with EDSS at baseline and follow-up, whereas *Agathobaculum* sp003481705 exhibited consistent inverse correlations. Additional taxa, including *Lachnospira* sp003451515, CAG-288 sp000437395, and *Coprococcus eutactus*, showed trend-level associations with EDSS at one or both visits. However, none of the taxon–EDSS associations remained significant after false discovery rate correction, and no associations with longitudinal changes in EDSS were identified (Table 4).

### 3.6. Predicted Functional Analysis

Additional exploratory analyses evaluated associations between bacterial taxa and clinical characteristics, including disability, baseline disease features, dietary characteristics, age, sex, and serum biomarkers. Several nominal associations were observed; however, none remained significant after correction for multiple testing and should therefore be considered hypothesis-generating, as no robust associations between individual taxa and neurological disability were identified (Supplementary Tables S5–S8).

Longitudinal analyses of Gut Metabolic Modules (GMMs) and Gut–Brain Modules (GBMs) identified nominal changes in selected pathways, including aspartate degradation II and isovaleric acid synthesis I, but none of these changes remained significant after FDR correction. Overall, these results indicate that the relative taxonomic stability observed over one year was accompanied by no robust evidence of systematic changes in predicted microbial functional potential (Supplementary Tables S9 and S10).

Moreover, we explored associations between predicted functional potential related to SCFA and tryptophan metabolism and EDSS or serum biomarkers.

A nominal positive correlation was observed between predicted functional potential for the propionate synthesis I pathway and NfL, suggesting a potential relationship between microbial metabolic potential and neuroaxonal injury; however, this association did not survive FDR correction. No robust associations were identified for the remaining SCFA- or tryptophan-related pathways with EDSS, GFAP, NfL, or S100B (Supplementary Tables S11 and S12). Accordingly, these exploratory findings do not provide evidence for a consistent relationship between predicted microbial metabolic potential and neurological disability or biomarkers.

## 4. Discussion

### 4.1. Gut Microbiota Stability and Disability Progression

Defining a healthy gut microbiota is challenging because of its substantial interindividual variability. Nevertheless, certain characteristics are generally considered hallmarks of a healthy microbiota, including greater microbial diversity and a higher abundance of butyrate-producing bacteria. Microbiota resilience refers to the ability of the microbial ecosystem to return to its original state following a perturbation and is therefore conceptually distinct from longitudinal stability, whereas dysbiosis arises when the baseline equilibrium cannot be re-established [8,9].

Microbial diversity, a measure of gut microbiota variability, encompasses both richness (the number of species) and evenness (the relative distribution of species). Greater microbial diversity is generally considered an indicator of ecosystem stability, although diversity alone does not establish ecological resilience. Alpha diversity reflects the diversity within a single sample, whereas beta diversity measures differences in microbial composition between samples [22].

Studies investigating the longitudinal dynamics of microbial diversity in MS are limited. A recent 2024 study including 58 PwMS found no significant differences in overall gut microbiota diversity between patients with and without disability progression. However, changes in specific taxa were associated with increases in the EDSS score, including higher abundances of *Alloprevotella*, *Prevotella-9*, and members of the *Rhodospirillales* order, together with reduced abundances of *Akkermansia*, *Lachnospiraceae*, and *Oscillospiraceae* [23]. A systematic review including more than 1700 PwMS, comprising predominantly cross-sectional studies, found no association between alpha or beta diversity and disease outcomes, such as disease activity or disability progression. In contrast, several individual taxa were associated with disability progression. Higher abundances of *Streptococcus*, *Clostridium nexile*, *Clostridium scindens*, and *Collinsella aerofaciens* were associated with worse outcomes, whereas higher abundances of *Actinobacteria*, *Bacteroidota*, and *Roseburia inulinivorans* were associated with more favorable outcomes. Overall, SCFA producers were associated with a more favorable disease course [1].

Our results are consistent with most studies in the literature, as alpha diversity did not change significantly over one year, as reflected in the Shannon, Simpson, and Chao indices. Beta diversity findings were more nuanced. At the cohort level, beta diversity remained stable over time, as indicated by the PERMANOVA test (*p* = 0.43). In contrast, individual patients exhibited moderate changes, reflecting a certain degree of microbiome reconfiguration (Bray–Curtis dissimilarity = 0.38). These findings suggest that microbial changes do not follow a common trajectory across individuals. Rather, each participant exhibits distinct microbiome dynamics, which collectively offset one another at the group level, resulting in the absence of significant overall compositional changes.

Although a few species showed nominal associations with disability measures, none remained significant after correction for multiple testing. *Agathobaculum* and Spyradocola merdavium have not yet been extensively studied [24,25]. These exploratory observations therefore do not provide evidence for specific taxon–disability relationships in our cohort and require validation in larger independent populations.

Patients with EDSS progression exhibited greater microbiome instability than those with stable or improved disability status. Although patients with EDSS progression showed numerically greater within-patient Bray–Curtis dissimilarity than those with stable or improved disability, this difference was not statistically significant (*p* = 0.328). This exploratory observation requires confirmation in larger longitudinal cohorts.

### 4.2. Gut Microbiome and MS Biomarkers

Numerous studies have highlighted the clinical relevance of serum biomarkers in MS. NfL is a well-established biomarker of inflammation and neuroaxonal injury that has been associated with disease activity, new MRI lesions, and clinical relapses [10]. Our results showed that NfL levels decreased over time in newly diagnosed PwMS, likely reflecting the gradual decline in the inflammatory component of the disease (*p* = 0.04).

In contrast, GFAP is considered a marker of disease progression, as its levels increase during the progressive phases of MS, when neurodegeneration and astrocytosis predominate. Although neurodegenerative processes are present from the earliest stages of the disease, they become increasingly prominent over time [11]. Consistent with this concept, GFAP levels increased over time in our cohort of PwMS (*p* = 0.001).

We also evaluated the potential of S100B as a biomarker in MS. Unlike NfL and GFAP, S100B has been less extensively studied. We did not observe any significant longitudinal changes in S100B levels. Although S100B is involved in the neuroinflammatory response, larger studies are needed to clarify its utility as a biomarker in MS [26].

Notably, none of the participants experienced a clinical relapse or received corticosteroid treatment during follow-up, reducing the potential influence of acute relapse-related inflammation or corticosteroid exposure on the observed longitudinal biomarker and microbiome profiles.

### 4.3. Longitudinal Microbiome Stability

An interesting finding of our study was the longitudinal stability of the gut microbiota, as reflected by alpha and beta diversity, despite dynamic changes in the serum biomarkers GFAP and NfL. This discrepancy may be explained by the distinct biological processes captured by these measures. While microbial diversity reflects the overall structure of the gut microbial ecosystem, serum biomarkers reflect ongoing neuroimmunological processes. Consequently, changes in disease course may occur without major alterations in the overall microbial community. These findings support longitudinal stability of overall gut microbial community structure in this cohort. However, stability across two timepoints should not be interpreted as evidence of microbiome resilience, which would require assessment of the microbial response to a defined perturbation and subsequent recovery toward the pre-perturbation state [27].

The one-year observation period should also be considered when interpreting this apparent stability. Microbiome changes related to disease evolution may occur over longer periods that cannot be captured by two sampling timepoints.

No statistically significant differences between the HE and ME groups were identified in longitudinal changes in clinical measures, serum biomarkers, diversity indices, or selected taxa in these exploratory analyses; however, treatment allocation was not randomized, and the groups were small and unequal in size. Therefore, a potential contribution of DMT exposure to the observed longitudinal changes cannot be excluded.

### 4.4. Functional Microbiome Redundancy

In a large study published in 2023, the authors generated an extensive collection of genome-scale metabolic models (GEMs) for the human gut microbiome by reconstructing the metabolic functions of 7302 microbial genomes. This approach enabled the prediction of microbial metabolic functions directly from metagenomic sequencing data. The authors demonstrated that microbial taxonomic composition does not necessarily correspond to metabolic function and highlighted functional redundancy as a key mechanism underlying microbiota resilience and stability. They concluded that taxonomic characterization alone provides insufficient insight into microbial activity and that functional capacity represents the more informative framework for microbiome research [28].

We explored the concept of functional redundancy, whereby a stable taxonomic composition enables the microbiota to adapt its metabolic functions in response to environmental or host-related factors [29]. Although several predicted functional pathways showed nominal longitudinal differences, none remained significant after correction for multiple testing. This reflects only nominal variation in predicted microbial functional potential, without evidence of statistically significant longitudinal change after multiple-testing correction.

Previous studies have shown that PwMS exhibit a microbial profile characterized by reduced SCFA production, particularly butyrate and propionate, representing one potential mechanism linking intestinal dysbiosis to neuroinflammation [2]. SCFAs are increasingly recognized as important mediators of immune regulation in MS. Butyrate exerts anti-inflammatory effects by modulating Treg cells and maintaining intestinal barrier integrity, whereas propionate also contributes to systemic immunomodulation [30]. Although several studies have investigated the relationship between SCFA-producing bacteria and clinical outcomes or disability, only a few have examined the direct association between SCFAs and serum biomarkers in MS [30,31].

The predicted functional analysis revealed no robust longitudinal changes in microbial functional potential after correction for multiple testing. Importantly, these metagenomically inferred pathways represent the genomic potential of the microbial community and not direct measurements of metabolic activity or metabolite concentrations. SCFA concentrations and tryptophan-derived metabolites were not directly quantified in the present study. Therefore, the observed nominal associations involving SCFA-related pathways should not be interpreted as evidence of altered SCFA production. We further explored these predicted metabolic pathways, and the predicted propionate synthesis potential showed a nominal correlation with NfL levels; however, this association did not remain significant after correction for multiple testing.

## 5. Conclusions

In this longitudinal study, the gut microbiome of patients with RRMS exhibited remarkable taxonomic stability despite significant longitudinal changes in serum neurological biomarkers. Exploratory taxonomic and predicted functional associations did not remain significant after correction for multiple testing and should therefore be considered hypothesis-generating. Overall, our findings support longitudinal gut microbiome stability over the one-year follow-up, but do not establish causal or mechanistic relationships between the gut microbiome, circulating neurological biomarkers, and clinical outcomes. Larger longitudinal studies with more frequent sampling and direct metabolomic assessment are needed to further investigate potential gut–brain relationships in MS.

Strengths and limitations: The major strength of our study is its longitudinal design in treatment-naïve patients, combined with the integration of gut microbiome analysis, clinical data, and serum biomarkers, as well as the comprehensive characterization of the microbiome using shotgun metagenomic sequencing.

However, several limitations should be acknowledged. The modest sample size represents an important limitation, reducing statistical power to detect small-to-moderate effects. Moreover, the large number of exploratory comparisons relative to the cohort size required multiple-testing correction, after which none of the nominal associations remained significant. Consequently, the absence of statistically significant associations should not be interpreted as evidence of the absence of biologically relevant relationships. These findings should therefore be regarded as hypothesis-generating and require validation in larger independent longitudinal cohorts.

Another important limitation is the lack of dietary standardization during follow-up. As this was an observational study designed to characterize microbiome dynamics under real-world conditions, no dietary intervention was imposed. Moreover, requiring dietary standardization in addition to the study eligibility criteria would have substantially limited recruitment feasibility. Although dietary habits were assessed at both visits using REAP-S, this instrument cannot fully capture dietary variability relevant to the gut microbiome. Therefore, residual dietary confounding cannot be excluded.

Because this study was exploratory in nature and had a limited sample size, subgroup and association analyses were considered hypothesis-generating, and non-significant findings should not be interpreted as evidence of the absence of an effect.

The one-year follow-up period, with microbiome assessment at two timepoints, represents an additional limitation. Longer follow-up periods with more frequent longitudinal sampling are needed.

## Figures and Tables

**Table 1:** Baseline characteristics of the study cohort (n = 29).

Variable	Value
Age, years	31 (27–48)
Female sex	18 (62.1%)
Smoking	7 (24.1%)
Dietary restrictions	5 (17.2%)
Disease duration, months	17 (6–64)
ARR	1.0 (0.39–1.0)
EDSS score	1.5 (1.0–2.0)
Treatment HE	20 (69.0%)
Treatment ME	9 (31.0%)

Continuous variables are presented as median (IQR); categorical variables are presented as n (%). Abbreviations: ARR = annualized relapse rate; EDSS = Expanded Disability Status Scale; HE = high efficacy treatment; ME = moderate-efficacy treatment; IQR = Interquartile Range.

**Table 2:** Evolution of clinical scores and serum neurological biomarkers.

Variable	V0	V1	*p*
EDSS score	1.5 (1.0–2.0)	1.5 (1.0–2.0)	0.114
T25FW, sec	5.91 (4.53–7.06)	5.36 (4.73–7.19)	0.495
9HPT dominant, sec	22.10 (17.70–25.38)	21.73 (18.95–25.47)	0.689
9HPT non-dominant, sec	22.60 (19.29–25.33)	22.25 (20.03–25.70)	0.658
SDMT	58 (50.5–76.5)	62 (46–86)	0.438
REAP total score	27 (25–29)	27 (24–29)	0.688
GFAP	79.74 (29.44–124.80)	104.00 (58.20–140.50)	0.001
NfL	26.12 (21.78–30.05)	24.68 (22.28–26.51)	0.040
S100B	0.00 (0.00–21.72)	8.77 (0.00–22.13)	0.091

Continuous variables are presented as median (IQR). Abbreviations: EDSS = Expanded Disability Status Scale, T25FW = 25-Foot Walk Test, 9HPT = 9-Hole Peg Test, SDMT = Symbol Digit Modalities Test, REAP = Rapid Eating Assessment for Participants, GFAP = glial fibrillary acidic protein, NfL = neurofilament light chain.

**Table 3:** Longitudinal changes in gut microbiome alpha diversity.

Metric	V0	V1	*p*
Shannon index	3.50 (2.97–3.79)	3.50 (3.14–3.79)	0.733
Simpson index	0.94 (0.88–0.96)	0.94 (0.90–0.95)	0.966
Chao1 richness	236 (196–266)	228 (188–285)	0.697

Comparison of alpha diversity indices between baseline (V0) and follow-up (V1).

**Table 4:** Correlations between LEfSe-derived taxa and EDSS at baseline, follow-up, and longitudinal change in EDSS.

Species	EDSS V0		EDSS V1		ΔEDSS	
	r	*p*	r	*p*	r	*p*
*Agathobaculum sp003481705*	−0.458	**0.013**	−0.391	**0.036**	0.096	0.621
*Spyradocola merdavium*	0.443	**0.016**	0.476	**0.009**	0.093	0.630
*Lachnospira sp003451515*	−0.357	0.058	−0.305	0.108	0.146	0.451
*CAG-288 sp000437395*	0.347	0.065	0.338	0.073	0.064	0.742
*Coprococcus eutactus*	0.347	0.065	0.343	0.069	0.211	0.272

Table 4 presents Spearman correlation coefficients between selected gut microbial species and EDSS at baseline, follow-up, and EDSS change over time. Abbreviations: r = Spearman’s rank correlation coefficient; V0 = baseline visit; V1 = follow-up visit; EDSS = Expanded Disability Status Scale. The bold for the initial statistically significant *p* values.

## Data Availability

The data and other information of this study are available from the corresponding author upon request.

## Supplementary Materials

The following supporting information can be downloaded at https://www.mdpi.com/article/10.3390/microorganisms14092080/s1. Figure S1: Baseline relative abundance of bacteria (V0); Figure S2: Alpha diversity Shannon index for both visits; Table S1: Ten most abundant bacterial genera at baseline (V0); Table S2: Relative abundance of bacterial phyla at baseline (V0); Table S3a: Exploratory comparison of longitudinal changes according to DMT efficacy category. Clinical, dietary, biomarker, and microbiome diversity outcomes; Table S3b: Exploratory comparison of longitudinal changes according to DMT efficacy category. Longitudinal changes in selected LEfSe-derived taxa according to DMT efficacy category; Table S4: The association between EDSS progression and changes in microbiome-derived metrics or serum biomarkers; Table S5: Exploratory correlations between LEfSe-derived taxa and clinical disability and diet assessments; Table S6: Exploratory correlations between LEfSe-derived taxa and disease characteristics and age; Table S7: Associations between selected LEfSe-derived taxa and sex; Table S8: Exploratory correlations between selected LEfSe-derived taxa and serum neurological biomarkers; Table S9: Longitudinal changes in gut metabolic modules (GMMs) between baseline and follow-up; Table S10: Longitudinal changes in gut–brain modules (GBMs) between baseline and follow-up; Table S11: Exploratory associations between SCFA-related pathways and clinical or biomarker variables; Table S12: Exploratory associations between tryptophan metabolism pathways and neurological biomarkers.
