# Supplementary material for: Longitudinal Stability of the Gut Microbiota in Relapsing–Remitting Multiple Sclerosis: A Prospective Observational Study
Source: Microorganisms. 2026 Sep 17;14(9):2080. doi: 10.3390/microorganisms14092080 (PMC13608969; doi:10.3390/microorganisms14092080)

SUPPLEMENTARY MATERIALS

Supplementary Table S1. Ten most abundant bacterial genera at baseline (V0).

Genus	Mean relative abundance (%)
Prevotella	12.22
Faecalibacterium	9.60
Phocaeicola	6.51
Bacteroides	5.04
Alistipes	3.88
Ruminococcus_E	3.45
Blautia_A	3.23
Agathobacter	2.86
Vescimonas	2.50
CAG-170	1.89

Supplementary Table S2. Relative abundance of bacterial phyla at baseline (V0).

Phylum	Mean relative abundance (%)
Bacillota_A	55.0
Bacteroidota	33.3
Pseudomonadota	3.4
Bacillota_C	2.3
Bacillota	1.8
Actinomycetota	1.6
Verrucomicrobiota	1.1
Cyanobacteriota	0.9
Desulfobacterota	0.3

Supplementary Table S3a. Exploratory comparison of longitudinal changes according to DMT efficacy category. Clinical, dietary, biomarker, and microbiome diversity outcomes

Variable	HE (n=20), median (IQR)	ME (n=9), median (IQR)	p-value	FDR
ΔShannon index	0.015 (−0.264–0.160)	0.194 (−0.185–0.272)	0.494	0.906
ΔSimpson index	0.002 (−0.026–0.019)	−0.004 (−0.012–0.013)	0.654	0.906
ΔChao1 richness	4.5 (−24.5–33.3)	2.0 (−27.0–26.0)	0.906	0.906
Bray–Curtis dissimilarity	0.363 (0.294–0.438)	0.479 (0.353–0.502)	0.114	0.749
ΔEDSS	0.0 (0.0–0.0)	0.0 (0.0–0.5)	0.802	0.906
ΔGFAP, pg/mL	15.87 (0.00–36.64)	17.07 (0.00–37.98)	0.850	0.906

ΔNfL, pg/mL	−0.41 (−3.35–0.31)	−2.10 (−8.10–0.90)	0.706	0.906
ΔS100B, pg/mL	0.00 (0.00–8.69)	10.53 (0.00–22.63)	0.115	0.749
ΔT25FW, s	0.24 (−0.75–1.25)	−0.30 (−0.92–0.72)	0.795	0.906
Δ9HPT dominant, s	0.45 (−1.20–1.61)	−0.49 (−2.35–1.01)	0.869	0.906
Δ9HPT non-dominant, s	−0.16 (−1.84–1.47)	1.20 (−0.15–3.90)	0.212	0.906
ΔSDMT	2.0 (−6.25–6.50)	3.0 (−2.00–5.00)	0.688	0.906
ΔREAP score	1.0 (0.00–3.25)	0.0 (−6.00–1.00)	0.365	0.906

Values are presented as median (IQR). Δ represents the within-participant change from baseline (V0) to follow-up (V1), calculated as V1−V0. Bray–Curtis dissimilarity represents within-participant microbiome dissimilarity between V0 and V1 and is therefore not a change score. Comparisons between the high-efficacy (HE) and moderate-efficacy (ME) treatment groups were performed using the Wilcoxon rank-sum test. FDR values were calculated using the Benjamini–Hochberg procedure across the 13 exploratory comparisons. No comparison was statistically significant before or after FDR correction.

Supplementary Table S3b. Exploratory comparison of longitudinal changes according to DMT efficacy category. Longitudinal changes in selected LEfSe-derived taxa according to DMT efficacy category

Species	HE (n=20), median Δ (IQR)	ME (n=9), median Δ (IQR)	p-value	FDR
Agathobaculum sp003481705	0 (−0.000282–0)	0 (0–0)	0.730	0.789
CAG-288 sp000437395	0 (0–0)	0 (0–0)	0.789	0.789
Coprococcus eutactus	0 (0–0)	0 (0–0)	0.629	0.789
Lachnospira sp003451515	0 (0–0)	0 (0–0)	0.721	0.789
Spyradocola merdavium	0 (−0.000066–0.000028)	0 (−0.000023–0.000394)	0.518	0.789

Δ represents the within-participant change in relative abundance from V0 to V1 (V1−V0). Comparisons between HE and ME groups were performed using the Wilcoxon rank-sum test. Benjamini–Hochberg FDR correction was applied across the five taxon-level comparisons.

Supplementary Table S4. The association between EDSS progression and changes in microbiome-derived metrics or serum biomarkers

Variable	Spearman rho	p-value
Bray–Curtis dissimilarity	0.321	0.090
ΔShannon diversity	0.144	0.457
ΔSimpson diversity	0.001	0.995
ΔChao1 richness	0.233	0.224
ΔGFAP	-0.063	0.745
ΔNfL	0.121	0.530
ΔS100B	0.040	0.835

Supplementary Table S5. Exploratory correlations between LEfSe-derived taxa and clinical disability and diet assessments

Species	Variable	Spearman rho	p	FDR
Anaerostipes hadrus_A	Digit Symbol Test	0.519	**0.004**	0.807
Duodenibacillus massiliensis	REAP score	-0.468	**0.010**	0.807
Agathobaculum sp003481705	EDSS	-0.458	**0.013**	0.807
Spyradocola merdavium	EDSS	0.443	**0.016**	0.807
Anaerostipes hadrus_A	9-Hole Peg Test, dominant hand	-0.431	**0.019**	0.807
Barnesiella intestinihominis	Digit Symbol Test	0.423	**0.022**	0.807
Lachnospira sp003451515	Digit Symbol Test	0.373	**0.046**	0.807
Anaerostipes hadrus_A	Timed 25-Foot Walk	-0.367	0.050	0.807
Agathobaculum sp003481705	Digit Symbol Test	0.357	0.057	0.807
Lachnospira sp003451515	EDSS	-0.357	0.058	0.807
CAG-288 sp000437395	Digit Symbol Test	-0.352	0.061	0.807
CAG-288 sp000437395	EDSS	0.347	0.065	0.807
Coprococcus eutactus	EDSS	0.347	0.065	0.807
UBA737 sp900549055	Digit Symbol Test	-0.346	0.066	0.807
Avimicrobium caecorum	Digit Symbol Test	0.345	0.066	0.807
Spyradocola merdavium	Timed 25-Foot Walk	0.344	0.068	0.807
Avimicrobium caecorum	9-Hole Peg Test, dominant hand	-0.338	0.073	0.807
Lachnospira sp003451515	REAP score	0.332	0.079	0.807
Phocaeicola sp000432735	Timed 25-Foot Walk	0.328	0.082	0.807
Faecalibacterium sp900758465	Timed 25-Foot Walk	0.325	0.086	0.807
Dorea_A longicatena_B	Digit Symbol Test	-0.322	0.088	0.807
Faecalibacterium sp900758465	EDSS	0.318	0.093	0.807
CAG-288 sp000437395	Timed 25-Foot Walk	0.317	0.094	0.807
CAG-433 sp000433675	Digit Symbol Test	-0.317	0.094	0.807
Victivallis sp900550905	REAP score	0.316	0.094	0.807
CAG-433 sp000433675	9-Hole Peg Test, non-dominant hand	-0.316	0.095	0.807
Dorea_A longicatena_B	Timed 25-Foot Walk	0.311	0.101	0.807
Ruthenibacterium lactatiformans	9-Hole Peg Test, non-dominant hand	0.31	0.102	0.807
CAG-302 sp000431795	9-Hole Peg Test, non-dominant hand	-0.307	0.105	0.807
Dialister sp000434475	REAP score	-0.307	0.105	0.807
Coprococcus eutactus	Digit Symbol Test	-0.304	0.109	0.807
Faecalibacterium sp900539945	EDSS	0.304	0.109	0.807
Egerieenecus merdigallinarum	9-Hole Peg Test, dominant hand	-0.3	0.114	0.807
Duodenibacillus intestinigallinarum	Digit Symbol Test	-0.299	0.115	0.807
Scatocola faecipullorum	Digit Symbol Test	0.299	0.115	0.807
Coprococcus_A catus	Digit Symbol Test	-0.295	0.121	0.807
Alistipes onderdonkii	EDSS	0.294	0.121	0.807
Faecalibacterium prausnitzii_E	9-Hole Peg Test, dominant hand	-0.294	0.122	0.807
Hungatella hathewayi	REAP score	0.292	0.125	0.807

Avimicrobium caecorum	Timed 25-Foot Walk	-0.291	0.126	0.807
Alectryocaccomicrobium faecavium	EDSS	0.291	0.126	0.807
Barnesiella intestinihominis	Timed 25-Foot Walk	-0.289	0.129	0.807
Parabacteroides merdae	9-Hole Peg Test, non-dominant hand	0.281	0.140	0.840
Butyribacter sp003524945	REAP score	0.276	0.147	0.862
Spyradocola merdavium	Digit Symbol Test	-0.274	0.150	0.862
Coprococcus eutactus	Timed 25-Foot Walk	0.267	0.162	0.862
Alistipes onderdonkii	Timed 25-Foot Walk	0.266	0.164	0.862
Alectryocaccomicrobium faecavium	Timed 25-Foot Walk	0.264	0.167	0.862
Victivallis sp900550905	9-Hole Peg Test, non-dominant hand	-0.262	0.170	0.862
Phocaeicola sp000432735	9-Hole Peg Test, non-dominant hand	-0.255	0.183	0.862
Lachnospira sp003451515	Timed 25-Foot Walk	-0.253	0.186	0.862
Flavonifractor plautii	Timed 25-Foot Walk	-0.252	0.188	0.862
Negativibacillus sp000435195	EDSS	0.251	0.189	0.862
Faecalibacterium sp900539945	9-Hole Peg Test, dominant hand	0.249	0.192	0.862
CAG-488 sp000434055	9-Hole Peg Test, non-dominant hand	-0.246	0.198	0.862
Duodenibacillus intestinigallinarum	EDSS	0.245	0.201	0.862
Spyradocola merdavium	9-Hole Peg Test, non-dominant hand	0.244	0.202	0.862
Anaerotardibacter sp000436075	REAP score	-0.241	0.207	0.862
Avimicrobium caecorum	REAP score	0.239	0.212	0.862
CAG-433 sp000433675	REAP score	0.239	0.212	0.862
Phascolarctobacterium faecium	Timed 25-Foot Walk	0.237	0.215	0.862
Bariatricus comes	REAP score	-0.236	0.217	0.862
CAG-302 sp000431795	Digit Symbol Test	-0.233	0.224	0.862
Faecalibacterium prausnitzii_F	Digit Symbol Test	0.231	0.229	0.862
Phocaeicola sp000432735	Digit Symbol Test	-0.227	0.237	0.862
Faecalibacterium prausnitzii_E	9-Hole Peg Test, non-dominant hand	-0.226	0.238	0.862
UMGS1865 sp900555785	Timed 25-Foot Walk	0.226	0.239	0.862
Faecalibacterium prausnitzii_E	Timed 25-Foot Walk	-0.226	0.239	0.862
Alectryocaccomicrobium faecavium	Digit Symbol Test	-0.226	0.239	0.862
UBA737 sp900549055	EDSS	0.225	0.240	0.862
Merdousia sp900545705	REAP score	-0.223	0.245	0.862
CAG-488 sp000434055	Digit Symbol Test	-0.22	0.252	0.862
Alistipes onderdonkii	9-Hole Peg Test, dominant hand	0.218	0.255	0.862
Faecalibacterium sp900539945	Timed 25-Foot Walk	0.218	0.257	0.862
Phocaeicola sp000432735	EDSS	0.217	0.258	0.862
Negativibacillus sp000435195	Timed 25-Foot Walk	0.216	0.260	0.862
Alistipes onderdonkii	REAP score	0.212	0.269	0.862
Anaerostipes hadrus_A	9-Hole Peg Test, non-dominant hand	-0.212	0.271	0.862
Egerieenecus merdigallinarum	EDSS	0.211	0.272	0.862
Faecalibacterium prausnitzii_F	EDSS	-0.208	0.278	0.862
Avimicrobium caecorum	9-Hole Peg Test, non-dominant hand	-0.208	0.279	0.862
Dialister sp000434475	Digit Symbol Test	0.204	0.290	0.862
Dorea_A longicatena_B	9-Hole Peg Test, non-dominant hand	0.203	0.291	0.862

Merdousia sp900545705	Timed 25-Foot Walk	0.2	0.297	0.862
Barnesiella intestinihominis	9-Hole Peg Test, dominant hand	-0.2	0.299	0.862
Lachnospira sp003451515	9-Hole Peg Test, dominant hand	-0.2	0.299	0.862
Dorea_A longicatena_B	EDSS	0.198	0.303	0.862
Alectryocaccomicrobium faecavium	9-Hole Peg Test, dominant hand	0.198	0.303	0.862
CAG-488 sp000434055	REAP score	0.197	0.305	0.862
Egerieenecus merdigallinarum	9-Hole Peg Test, non-dominant hand	-0.197	0.307	0.862
Dorea_A longicatena_B	9-Hole Peg Test, dominant hand	0.196	0.307	0.862
Phascolarctobacterium faecium	9-Hole Peg Test, dominant hand	0.192	0.318	0.862
CAG-488 sp000434055	Timed 25-Foot Walk	0.187	0.331	0.862
UBA737 sp900549055	Timed 25-Foot Walk	0.181	0.348	0.862
CAG-433 sp000433675	9-Hole Peg Test, dominant hand	-0.181	0.348	0.862
Alectryocaccomicrobium faecavium	REAP score	0.18	0.349	0.862
Anaerostipes hadrus_A	EDSS	-0.179	0.351	0.862
Agathobaculum sp003481705	Timed 25-Foot Walk	-0.178	0.355	0.862
Faecalibacterium prausnitzii_F	Timed 25-Foot Walk	-0.178	0.355	0.862
Faecalibacterium prausnitzii_F	9-Hole Peg Test, non-dominant hand	-0.177	0.359	0.862
Agathobaculum sp003481705	9-Hole Peg Test, dominant hand	-0.176	0.360	0.862
Agathobaculum sp003481705	REAP score	0.175	0.364	0.862
Bariatricus comes	9-Hole Peg Test, non-dominant hand	0.172	0.371	0.862
Agathobaculum sp003481705	9-Hole Peg Test, non-dominant hand	-0.172	0.372	0.862
Negativibacillus sp000435195	REAP score	0.168	0.384	0.862
Victivallis sp900550905	EDSS	0.166	0.391	0.862
Victivallis sp900550905	Digit Symbol Test	-0.164	0.396	0.862
Hungatella hathewayi	EDSS	0.159	0.409	0.862
UMGS1865 sp900555785	REAP score	0.159	0.410	0.862
Merdousia sp900760665	REAP score	0.159	0.410	0.862
Prevotella massiliensis	REAP score	0.159	0.410	0.862
Coprobacter fastidiosus	Timed 25-Foot Walk	-0.158	0.412	0.862
UBA737 sp900549055	REAP score	-0.158	0.412	0.862
Merdousia sp900545705	9-Hole Peg Test, non-dominant hand	0.156	0.419	0.862
Negativibacillus sp000435195	9-Hole Peg Test, non-dominant hand	-0.154	0.424	0.862
CAG-288 sp000437395	REAP score	0.153	0.429	0.862
Duodenibacillus intestinigallinarum	Timed 25-Foot Walk	0.153	0.429	0.862
Phocaeicola sp000432735	9-Hole Peg Test, dominant hand	-0.152	0.430	0.862
UMGS1865 sp900555785	EDSS	0.151	0.434	0.862
CAG-433 sp000433675	EDSS	0.151	0.434	0.862
Spyradocola merdavium	9-Hole Peg Test, dominant hand	0.149	0.440	0.862
UBA1394 sp900538575	9-Hole Peg Test, dominant hand	0.148	0.444	0.862
Faecalibacterium prausnitzii_E	REAP score	-0.148	0.444	0.862
Butyribacter sp003524945	EDSS	0.145	0.454	0.862
Parabacteroides merdae	EDSS	0.143	0.460	0.862
COE1 sp001916965	Digit Symbol Test	0.143	0.460	0.862
Phascolarctobacterium faecium	REAP score	0.142	0.463	0.862

Duodenibacillus massiliensis	9-Hole Peg Test, dominant hand	-0.141	0.465	0.862
Coprobacter fastidiosus	REAP score	-0.141	0.465	0.862
Merdousia sp900545705	9-Hole Peg Test, dominant hand	0.138	0.474	0.862
UBA1394 sp900538575	Timed 25-Foot Walk	0.137	0.479	0.862
CAG-302 sp000431795	Timed 25-Foot Walk	0.136	0.482	0.862
Faecalibacterium prausnitzii_E	Digit Symbol Test	0.136	0.483	0.862
UMGS1865 sp900555785	9-Hole Peg Test, non-dominant hand	0.136	0.483	0.862
Merdousia sp900760665	9-Hole Peg Test, non-dominant hand	-0.136	0.483	0.862
Prevotella massiliensis	9-Hole Peg Test, non-dominant hand	-0.136	0.483	0.862
Dialister sp000434475	Timed 25-Foot Walk	-0.136	0.483	0.862
Dialister sp000434475	9-Hole Peg Test, dominant hand	-0.136	0.483	0.862
UMGS1865 sp900555785	9-Hole Peg Test, dominant hand	0.136	0.483	0.862
Faecalibacterium sp900758465	9-Hole Peg Test, dominant hand	0.135	0.484	0.862
UBA1394 sp900538575	9-Hole Peg Test, non-dominant hand	0.132	0.494	0.870
CAG-488 sp000434055	EDSS	-0.131	0.497	0.870
Avimicrobium caecorum	EDSS	-0.13	0.500	0.870
Parabacteroides merdae	9-Hole Peg Test, dominant hand	0.127	0.511	0.871
CAG-488 sp000434055	9-Hole Peg Test, dominant hand	-0.126	0.514	0.871
Anaerotardibacter sp000436075	Digit Symbol Test	-0.126	0.515	0.871
Anaerostipes hadrus_A	REAP score	-0.126	0.515	0.871
Scatocola faecipullorum	9-Hole Peg Test, dominant hand	-0.124	0.520	0.871
Merdousia sp900545705	Digit Symbol Test	-0.124	0.522	0.871
Coprococcus eutactus	REAP score	0.124	0.523	0.871
Flavonifractor plautii	9-Hole Peg Test, dominant hand	-0.123	0.525	0.871
Victivallis sp900550905	Timed 25-Foot Walk	0.122	0.527	0.871
Dorea_A longicatena_B	REAP score	-0.12	0.535	0.878
Faecalibacterium sp900539945	9-Hole Peg Test, non-dominant hand	0.12	0.537	0.878
Alistipes onderdonkii	9-Hole Peg Test, non-dominant hand	0.118	0.541	0.880
Parabacteroides merdae	REAP score	0.116	0.548	0.880
Dialister sp000434475	9-Hole Peg Test, non-dominant hand	-0.113	0.559	0.880
Bariatricus comes	Digit Symbol Test	-0.112	0.561	0.880
Phascolarctobacterium faecium	9-Hole Peg Test, non-dominant hand	0.112	0.564	0.880
Lachnospira sp003451515	9-Hole Peg Test, non-dominant hand	-0.11	0.570	0.885
Faecalibacterium sp900539945	REAP score	-0.108	0.579	0.893
Duodenibacillus massiliensis	Digit Symbol Test	-0.105	0.587	0.900
UBA1394 sp900538575	EDSS	0.104	0.590	0.900
Flavonifractor plautii	REAP score	-0.104	0.592	0.900
Victivallis sp900550905	9-Hole Peg Test, dominant hand	-0.102	0.600	0.908
COE1 sp001916965	EDSS	-0.1	0.607	0.913
Coprobacter fastidiosus	Digit Symbol Test	0.095	0.624	0.916
Parabacteroides merdae	Timed 25-Foot Walk	-0.094	0.626	0.916
Hungatella hathewayi	Digit Symbol Test	0.093	0.630	0.916
Coprococcus_A catus	9-Hole Peg Test, non-dominant hand	0.093	0.630	0.916
Duodenibacillus massiliensis	9-Hole Peg Test, non-dominant hand	0.092	0.635	0.916

Duodenibacillus massiliensis	EDSS	-0.092	0.636	0.916
Alectryocaccomicrobium faecavium	9-Hole Peg Test, non-dominant hand	0.091	0.640	0.916
Negativibacillus sp000435195	Digit Symbol Test	-0.09	0.641	0.916
CAG-302 sp000431795	REAP score	0.089	0.645	0.916
UBA737 sp900549055	9-Hole Peg Test, non-dominant hand	-0.089	0.645	0.916
Faecalibacterium sp900758465	Digit Symbol Test	-0.089	0.647	0.916
Hungatella hathewayi	Timed 25-Foot Walk	0.088	0.648	0.916
Flavonifractor plautii	Digit Symbol Test	-0.082	0.672	0.923
Hungatella hathewayi	9-Hole Peg Test, non-dominant hand	0.082	0.674	0.923
CAG-288 sp000437395	9-Hole Peg Test, dominant hand	0.082	0.674	0.923
Alistipes onderdonkii	Digit Symbol Test	-0.08	0.678	0.923
UBA1394 sp900538575	Digit Symbol Test	0.08	0.681	0.923
Faecalibacterium sp900758465	REAP score	-0.08	0.682	0.923
Coprobacter fastidiosus	9-Hole Peg Test, dominant hand	-0.079	0.682	0.923
UBA1394 sp900538575	REAP score	0.079	0.684	0.923
COE1 sp001916965	9-Hole Peg Test, dominant hand	0.078	0.688	0.923
Butyribacter sp003524945	Timed 25-Foot Walk	0.077	0.690	0.923
Bariatricus comes	EDSS	0.076	0.694	0.923
Faecalibacterium prausnitzii_F	9-Hole Peg Test, dominant hand	-0.073	0.705	0.923
Ruthenibacterium lactatiformans	Timed 25-Foot Walk	-0.07	0.719	0.923
Butyribacter sp003524945	Digit Symbol Test	0.068	0.726	0.923
Merdousia sp900760665	Digit Symbol Test	-0.068	0.727	0.923
Prevotella massiliensis	Digit Symbol Test	-0.068	0.727	0.923
Scatocola faecipullorum	EDSS	0.067	0.728	0.923
Barnesiella intestinihominis	REAP score	0.067	0.730	0.923
Coprococcus_A catus	Timed 25-Foot Walk	0.067	0.731	0.923
Egerieenecus merdigallinarum	REAP score	0.067	0.731	0.923
Hungatella hathewayi	9-Hole Peg Test, dominant hand	-0.064	0.740	0.923
CAG-302 sp000431795	9-Hole Peg Test, dominant hand	-0.064	0.740	0.923
Faecalibacterium prausnitzii_F	REAP score	-0.062	0.748	0.923
COE1 sp001916965	Timed 25-Foot Walk	0.062	0.750	0.923
Coprobacter fastidiosus	EDSS	-0.06	0.756	0.923
Coprococcus eutactus	9-Hole Peg Test, dominant hand	0.059	0.759	0.923
Coprococcus_A catus	REAP score	-0.059	0.762	0.923
UBA737 sp900549055	9-Hole Peg Test, dominant hand	0.058	0.764	0.923
Butyribacter sp003524945	9-Hole Peg Test, dominant hand	0.058	0.764	0.923
Spyradocola merdavium	REAP score	0.057	0.768	0.923
Anaerotardibacter sp000436075	Timed 25-Foot Walk	0.056	0.772	0.923
Parabacteroides merdae	Digit Symbol Test	-0.056	0.773	0.923
Coprobacter fastidiosus	9-Hole Peg Test, non-dominant hand	-0.054	0.780	0.923
Merdousia sp900545705	EDSS	-0.052	0.789	0.923
Barnesiella intestinihominis	EDSS	0.051	0.793	0.923
Ruthenibacterium lactatiformans	REAP score	-0.051	0.793	0.923
Barnesiella intestinihominis	9-Hole Peg Test, non-dominant hand	-0.051	0.795	0.923

Scatocola faecipullorum	Timed 25-Foot Walk	0.05	0.796	0.923
CAG-288 sp000437395	9-Hole Peg Test, non-dominant hand	0.049	0.801	0.923
Faecalibacterium sp900758465	9-Hole Peg Test, non-dominant hand	-0.048	0.804	0.923
Bariatricus comes	Timed 25-Foot Walk	-0.047	0.807	0.923
COE1 sp001916965	9-Hole Peg Test, non-dominant hand	-0.047	0.810	0.923
Ruthenibacterium lactatiformans	EDSS	0.047	0.810	0.923
Bariatricus comes	9-Hole Peg Test, dominant hand	-0.047	0.810	0.923
Anaerotardibacter sp000436075	9-Hole Peg Test, non-dominant hand	0.046	0.813	0.923
Flavonifractor plautii	9-Hole Peg Test, non-dominant hand	0.046	0.813	0.923
Ruthenibacterium lactatiformans	9-Hole Peg Test, dominant hand	0.045	0.815	0.923
CAG-433 sp000433675	Timed 25-Foot Walk	0.045	0.816	0.923
Egerieenecus merdigallinarum	Timed 25-Foot Walk	-0.044	0.821	0.925
Coprococcus_A catus	9-Hole Peg Test, dominant hand	-0.041	0.833	0.934
Phascolarctobacterium faecium	EDSS	0.041	0.834	0.934
Duodenibacillus intestinigallinarum	9-Hole Peg Test, dominant hand	0.038	0.844	0.941
CAG-302 sp000431795	EDSS	0.037	0.851	0.942
Duodenibacillus intestinigallinarum	REAP score	0.036	0.852	0.942
Coprococcus eutactus	9-Hole Peg Test, non-dominant hand	-0.035	0.858	0.942
Anaerotardibacter sp000436075	9-Hole Peg Test, dominant hand	0.034	0.863	0.942
Negativibacillus sp000435195	9-Hole Peg Test, dominant hand	-0.03	0.877	0.950
Scatocola faecipullorum	REAP score	-0.026	0.893	0.964
UMGS1865 sp900555785	Digit Symbol Test	0.023	0.907	0.969
Merdousia sp900760665	9-Hole Peg Test, dominant hand	0.023	0.907	0.969
Prevotella massiliensis	9-Hole Peg Test, dominant hand	0.023	0.907	0.969
Egerieenecus merdigallinarum	Digit Symbol Test	-0.021	0.916	0.973
Flavonifractor plautii	EDSS	0.02	0.918	0.973
Phascolarctobacterium faecium	Digit Symbol Test	0.015	0.938	0.982
Faecalibacterium sp900539945	Digit Symbol Test	-0.013	0.946	0.982
COE1 sp001916965	REAP score	-0.013	0.947	0.982
Duodenibacillus intestinigallinarum	9-Hole Peg Test, non-dominant hand	-0.012	0.952	0.982
Dialister sp000434475	EDSS	-0.012	0.952	0.982
Faecalibacterium prausnitzii_E	EDSS	-0.012	0.952	0.982
Merdousia sp900760665	EDSS	-0.012	0.952	0.982
Prevotella massiliensis	EDSS	-0.012	0.952	0.982
Ruthenibacterium lactatiformans	Digit Symbol Test	-0.01	0.960	0.985
Scatocola faecipullorum	9-Hole Peg Test, non-dominant hand	-0.009	0.962	0.985
Butyribacter sp003524945	9-Hole Peg Test, non-dominant hand	0.007	0.972	0.992
Phocaeicola sp000432735	REAP score	0.005	0.979	0.994
Coprococcus_A catus	EDSS	0.004	0.982	0.994
Anaerotardibacter sp000436075	EDSS	-0.004	0.984	0.994
Duodenibacillus massiliensis	Timed 25-Foot Walk	-0.001	0.995	1.000
Merdousia sp900760665	Timed 25-Foot Walk	0	1.000	1.000
Prevotella massiliensis	Timed 25-Foot Walk	0	1.000	1.000

Nominal p values < 0.05 are shown in bold; none should be interpreted without considering FDR correction.

Supplementary Table S6. Exploratory correlations between LEfSe-derived taxa and disease characteristics and age.

**Species**	**Variable**	**Spearman rho**	**p**	**FDR**
Agathobaculum sp003481705	Disease duration	-0.507	**0.005**	0.344
Anaerostipes hadrus_A	Disease duration	-0.474	**0.009**	0.344
Dorea_A longicatena_B	Disease duration	0.444	**0.016**	0.344
Avimicrobium caecorum	Relapses/year	0.443	**0.016**	0.344
Anaerostipes hadrus_A	Relapses/year	0.389	**0.037**	0.640
Negativibacillus sp000435195	Age	0.371	**0.048**	0.807
Victivallis sp900550905	Age	0.361	0.054	0.807
CAG-288 sp000437395	Age	0.354	0.060	0.807
Phocaeicola sp000432735	Age	0.331	0.080	0.807
Dorea_A longicatena_B	Relapses/year	-0.326	0.085	0.933
Alistipes onderdonkii	Age	0.324	0.086	0.807
UMGS1865 sp900555785	Age	0.317	0.094	0.807
Agathobaculum sp003481705	Relapses/year	0.305	0.108	0.933
Avimicrobium caecorum	Disease duration	-0.295	0.120	0.933
Phascolarctobacterium faecium	Age	0.285	0.134	0.820
Flavonifractor plautii	Relapses/year	0.283	0.136	0.933
Coprococcus eutactus	Age	0.268	0.160	0.862
Negativibacillus sp000435195	Relapses/year	-0.262	0.170	0.933
Alectryocaccomicrobium faecavium	Age	0.26	0.174	0.862
Faecalibacterium prausnitzii_E	Relapses/year	0.256	0.181	0.933
Faecalibacterium prausnitzii_F	Age	-0.252	0.187	0.862
Alistipes onderdonkii	Relapses/year	-0.251	0.188	0.933
Dialister sp000434475	Disease duration	-0.249	0.193	0.933
Egerieenecus merdigallinarum	Age	0.248	0.194	0.862
Hungatella hathewayi	Relapses/year	0.247	0.197	0.933
Agathobaculum sp003481705	Age	-0.242	0.207	0.862
Lachnospira sp003451515	Relapses/year	0.239	0.212	0.933
Phascolarctobacterium faecium	Relapses/year	-0.238	0.213	0.933
Dialister sp000434475	Age	-0.226	0.238	0.862
Bariatricus comes	Relapses/year	0.225	0.240	0.933
Parabacteroides merdae	Disease duration	0.223	0.244	0.933
CAG-288 sp000437395	Disease duration	0.221	0.250	0.933
Alistipes onderdonkii	Disease duration	0.215	0.262	0.933
Scatocola faecipullorum	Relapses/year	-0.214	0.264	0.933
Faecalibacterium sp900539945	Relapses/year	-0.214	0.266	0.933
Butyribacter sp003524945	Relapses/year	-0.21	0.274	0.933
UMGS1865 sp900555785	Relapses/year	-0.209	0.276	0.933
CAG-433 sp000433675	Age	0.204	0.289	0.862
Faecalibacterium prausnitzii_E	Disease duration	-0.203	0.290	0.933

Negativibacillus sp000435195	Disease duration	0.203	0.292	0.933
CAG-488 sp000434055	Age	0.201	0.295	0.862
CAG-302 sp000431795	Age	0.197	0.305	0.862
Faecalibacterium prausnitzii_F	Disease duration	-0.19	0.324	0.933
Faecalibacterium sp900758465	Age	0.184	0.339	0.862
Anaerotardibacter sp000436075	Disease duration	0.184	0.339	0.933
CAG-288 sp000437395	Relapses/year	-0.183	0.342	0.933
Butyribacter sp003524945	Age	0.182	0.345	0.862
UMGS1865 sp900555785	Disease duration	0.181	0.348	0.933
Barnesiella intestinihominis	Age	-0.177	0.358	0.862
Faecalibacterium prausnitzii_E	Age	-0.17	0.379	0.862
Ruthenibacterium lactatiformans	Age	-0.168	0.383	0.862
Spyradocola merdavium	Age	0.168	0.383	0.862
Anaerostipes hadrus_A	Age	-0.164	0.394	0.862
Anaerotardibacter sp000436075	Relapses/year	-0.163	0.400	0.933
Dorea_A longicatena_B	Age	0.16	0.406	0.862
Ruthenibacterium lactatiformans	Relapses/year	0.159	0.409	0.933
Parabacteroides merdae	Age	0.158	0.413	0.862
Coprobacter fastidiosus	Age	-0.157	0.417	0.862
Lachnospira sp003451515	Disease duration	-0.153	0.428	0.933
Lachnospira sp003451515	Age	-0.152	0.432	0.862
CAG-302 sp000431795	Relapses/year	-0.15	0.438	0.933
Parabacteroides merdae	Relapses/year	-0.148	0.444	0.933
Coprococcus eutactus	Relapses/year	-0.142	0.463	0.933
UBA1394 sp900538575	Age	0.141	0.464	0.862
Phocaeicola sp000432735	Disease duration	0.14	0.468	0.933
Scatocola faecipullorum	Age	0.14	0.468	0.862
Coprococcus eutactus	Disease duration	0.137	0.479	0.933
COE1 sp001916965	Age	-0.131	0.499	0.870
Merdousia sp900545705	Disease duration	0.127	0.510	0.933
Merdousia sp900760665	Disease duration	0.124	0.520	0.933
Prevotella massiliensis	Disease duration	0.124	0.520	0.933
Faecalibacterium sp900758465	Relapses/year	-0.122	0.529	0.933
CAG-488 sp000434055	Disease duration	-0.118	0.541	0.933
Faecalibacterium prausnitzii_F	Relapses/year	0.118	0.541	0.933
Phascolarctobacterium faecium	Disease duration	0.113	0.558	0.933
Merdousia sp900760665	Age	0.113	0.559	0.880
Prevotella massiliensis	Age	0.113	0.559	0.880
Flavonifractor plautii	Age	-0.113	0.560	0.880
Avimicrobium caecorum	Age	-0.113	0.561	0.880
Duodenibacillus massiliensis	Disease duration	0.112	0.563	0.933
Barnesiella intestinihominis	Disease duration	-0.112	0.564	0.933
Spyradocola merdavium	Disease duration	0.11	0.570	0.933
UBA1394 sp900538575	Relapses/year	-0.107	0.580	0.933

Merdousia sp900545705	Relapses/year	-0.097	0.615	0.933
Victivallis sp900550905	Disease duration	-0.097	0.616	0.933
Alectryocaccomicrobium faecavium	Relapses/year	-0.096	0.619	0.933
COE1 sp001916965	Disease duration	-0.094	0.628	0.933
Duodenibacillus intestinigallinarum	Age	0.093	0.631	0.916
Merdousia sp900760665	Relapses/year	-0.093	0.631	0.933
Prevotella massiliensis	Relapses/year	-0.093	0.631	0.933
Hungatella hathewayi	Disease duration	-0.082	0.674	0.933
Faecalibacterium sp900758465	Disease duration	0.08	0.679	0.933
UBA1394 sp900538575	Disease duration	0.08	0.681	0.933
Egerieenecus merdigallinarum	Relapses/year	0.078	0.689	0.933
CAG-302 sp000431795	Disease duration	-0.074	0.702	0.933
Spyradocola merdavium	Relapses/year	-0.073	0.706	0.933
Coprococcus_A catus	Disease duration	0.073	0.706	0.933
Duodenibacillus massiliensis	Relapses/year	-0.072	0.710	0.933
Bariatricus comes	Age	-0.071	0.713	0.923
Scatocola faecipullorum	Disease duration	0.07	0.716	0.933
Dialister sp000434475	Relapses/year	0.07	0.719	0.933
CAG-433 sp000433675	Relapses/year	0.07	0.719	0.933
COE1 sp001916965	Relapses/year	-0.067	0.729	0.933
Phocaeicola sp000432735	Relapses/year	-0.065	0.739	0.933
Duodenibacillus massiliensis	Age	0.064	0.743	0.923
Duodenibacillus intestinigallinarum	Disease duration	0.062	0.748	0.933
UBA737 sp900549055	Age	0.056	0.773	0.923
Coprococcus_A catus	Age	0.054	0.779	0.923
Alectryocaccomicrobium faecavium	Disease duration	0.054	0.781	0.952
Hungatella hathewayi	Age	-0.054	0.781	0.923
Egerieenecus merdigallinarum	Disease duration	-0.051	0.793	0.952
CAG-433 sp000433675	Disease duration	-0.045	0.816	0.952
UBA737 sp900549055	Relapses/year	0.045	0.817	0.952
Ruthenibacterium lactatiformans	Disease duration	-0.043	0.826	0.952
CAG-488 sp000434055	Relapses/year	0.042	0.830	0.952
Merdousia sp900545705	Age	0.035	0.856	0.942
Coprobacter fastidiosus	Disease duration	0.035	0.857	0.958
Butyribacter sp003524945	Disease duration	0.035	0.858	0.958
Faecalibacterium sp900539945	Age	0.033	0.864	0.942
Anaerotardibacter sp000436075	Age	-0.032	0.870	0.945
UBA737 sp900549055	Disease duration	0.024	0.900	0.969
Flavonifractor plautii	Disease duration	-0.019	0.922	0.969
Coprobacter fastidiosus	Relapses/year	0.018	0.925	0.969
Coprococcus_A catus	Relapses/year	-0.018	0.928	0.969
Duodenibacillus intestinigallinarum	Relapses/year	-0.017	0.929	0.969
Victivallis sp900550905	Relapses/year	-0.015	0.938	0.969
Barnesiella intestinihominis	Relapses/year	-0.013	0.946	0.969

Bariatricus comes	Disease duration	-0.007	0.973	0.975
Faecalibacterium sp900539945	Disease duration	-0.006	0.975	0.975

Nominal p values < 0.05 are shown in bold; none should be interpreted without considering FDR correction.

Supplementary Table S7. Associations between selected LEfSe-derived taxa and sex

Species	p-value	Median_F	Median_M	Variable
Agathobaculum sp003481705	0.416	0	0	Sex
CAG-288 sp000437395	0.447	0	0	Sex
Coprococcus eutactus	0.759	0	0	Sex
Spyradocola merdavium	0.886	1.15e-05	2.50e-05	Sex

Supplementary Table S8. Exploratory correlations between selected LEfSe-derived taxa and serum neurological biomarkers.

Species	Biomarker	Spearman rho	p	FDR
CAG-288 sp000437395	NfL	0.37	**0.049**	0.432
Coprococcus eutactus	NfL	0.357	0.058	0.432
CAG-288 sp000437395	S100B	-0.289	0.129	0.524
Agathobaculum sp003481705	NfL	-0.238	0.214	0.524
Spyradocola merdavium	GFAP	-0.237	0.215	0.524
Lachnospira sp003451515	S100B	-0.232	0.227	0.524
Coprococcus eutactus	GFAP	0.223	0.245	0.524
Spyradocola merdavium	NfL	0.205	0.286	0.535
Spyradocola merdavium	S100B	-0.112	0.562	0.871
CAG-288 sp000437395	GFAP	-0.099	0.609	0.871
Coprococcus eutactus	S100B	0.091	0.639	0.871
Agathobaculum sp003481705	S100B	0.063	0.745	0.926
Agathobaculum sp003481705	GFAP	0.032	0.869	0.926
Lachnospira sp003451515	GFAP	0.026	0.894	0.926
Lachnospira sp003451515	NfL	0.018	0.926	0.926

Supplementary Table S9. Longitudinal changes in gut metabolic modules (GMMs) between baseline and follow-up.

Pathway/module	p-value	FDR
aspartate degradation II	**0.006**	0.617
Bifidobacterium shunt	**0.024**	0.891
urea degradation	0.080	0.891
xylose degradation	0.096	0.891
sucrose degradation II	0.110	0.891
propionate production II	0.121	0.891
pyruvate:formate lyase	0.137	0.891
lactose and galactose degradation	0.148	0.891

Entner-Doudoroff pathway	0.149	0.891
propionate production III	0.163	0.891
melibiose degradation	0.169	0.891
tyrosine degradation II	0.176	0.891
alanine degradation II	0.183	0.891
galacturonate degradation I	0.183	0.891
lysine degradation II	0.221	0.891
pentose phosphate pathway (oxidative phase)	0.221	0.891
sucrose degradation I	0.221	0.891
fucose degradation	0.230	0.891
glutamate degradation II	0.230	0.891
phenylalanine degradation	0.235	0.891
trehalose degradation	0.238	0.891
sulfate reduction (dissimilatory)	0.250	0.891
cysteine degradation I	0.256	0.891
mucin degradation	0.256	0.891
propionate production I	0.259	0.891
alanine degradation I	0.265	0.891
cysteine degradation II	0.265	0.891
4-aminobutyrate degradation	0.275	0.891
histidine degradation	0.284	0.891
threonine degradation II	0.284	0.891
glutamine degradation I	0.294	0.891
methionine degradation I	0.304	0.891
butyrate production I	0.314	0.891
methanogenesis - methyl-coM	0.321	0.891
methanol conversion	0.331	0.891
glycerol degradation II	0.336	0.891
lysine degradation I	0.336	0.891
pectin degradation I	0.336	0.891
triacylglycerol degradation	0.344	0.891
fructan degradation	0.358	0.891
lactose degradation	0.358	0.891
lactate consumption I	0.381	0.891
hydrogen metabolism	0.393	0.891
butyrate production II	0.405	0.891
acetyl-CoA to crotonyl-CoA	0.417	0.891
tryptophan degradation	0.429	0.891
formate conversion	0.432	0.891
glutamate degradation I	0.439	0.891
glycerol degradation I	0.442	0.891
fructose degradation	0.468	0.891
pectine degradation II	0.468	0.891
starch degradation	0.468	0.891

lactate production	0.481	0.891
succinate conversion to propionate	0.493	0.891
methanogenesis from carbon dioxide	0.508	0.891
acetate to acetyl-CoA	0.508	0.891
anaerobic fatty acid beta-oxidation	0.508	0.891
proline degradation	0.508	0.891
glycerol degradation III	0.521	0.891
glyoxylate bypass	0.522	0.891
nitrate reduction (dissimilatory)	0.536	0.891
putrescine degradation	0.536	0.891
lactaldehyde degradation	0.565	0.899
serine degradation	0.565	0.899
arginine degradation IV	0.579	0.899
lactate consumption II	0.594	0.899
arginine degradation V	0.609	0.899
glutamine degradation II	0.609	0.899
glycine degradation	0.624	0.899
asparagine degradation	0.639	0.899
maltose degradation	0.639	0.899
pyruvate:ferredoxin oxidoreductase	0.639	0.899
valine degradation I	0.651	0.899
arabinoxylan degradation	0.654	0.899
galactose degradation	0.654	0.899
arginine degradation II	0.670	0.903
tyrosine degradation I	0.686	0.903
ethanol production II	0.701	0.903
rhamnose degradation	0.701	0.903
succinate consumption	0.701	0.903
mannose degradation	0.717	0.912
threonine degradation I	0.733	0.919
ethanol production I	0.749	0.919
isoleucine degradation	0.749	0.919
arabinose degradation	0.766	0.921
NADH:ferredoxin oxidoreductase	0.769	0.921
pyruvate dehydrogenase complex	0.779	0.923
succinate production	0.810	0.948
acetyl-CoA to acetate	0.831	0.962
leucine degradation	0.914	1.000
glycolysis (pay-off phase)	0.915	1.000
pentose phosphate pathway (non-oxidative branch)	0.932	1.000
ribose degradation	0.932	1.000
allose degradation	0.949	1.000
glutamate degradation III	0.966	1.000
glycolysis (preparatory phase)	0.966	1.000

homoacetogenesis	0.966	1.000
methionine degradation II	0.966	1.000
cysteine biosynthesis/homocysteine degradation	0.996	1.000
arginine degradation I	1.000	1.000
aspartate degradation I	1.000	1.000
arginine degradation III	1.000	1.000
galacturonate degradation II	1.000	1.000

Nominal p values < 0.05 are shown in bold; none should be interpreted without considering FDR correction.

Supplementary Table S10. Longitudinal changes in gut-brain modules (GBMs) between baseline and follow-up.

Pathway/module	p	FDR
Isovaleric acid synthesis I (KADH pathway)	**0.005**	0.256
Dopamine degradation	**0.025**	0.623
p-Cresol synthesis	0.066	0.778
Quinolinic acid degradation	0.076	0.778
Propionate synthesis II	0.121	0.778
g-Hydroxybutyric acid (GHB) degradation	0.132	0.778
Propionate synthesis III	0.163	0.778
Glutamate synthesis I	0.176	0.778
Quinolinic acid synthesis	0.198	0.778
GABA synthesis III	0.230	0.778
Kynurenine degradation	0.238	0.778
DOPAC synthesis	0.258	0.778
Propionate synthesis I	0.269	0.778
Histamine degradation	0.304	0.778
p-Cresol degradation	0.309	0.778
Butyrate synthesis I	0.314	0.778
Inositol degradation	0.314	0.778
GABA synthesis I	0.352	0.778
Butyrate synthesis II	0.405	0.778
Acetate degradation	0.417	0.778
Tryptophan synthesis	0.417	0.778
Cortisol degradation	0.429	0.778
GABA degradation	0.429	0.778
Tryptophan degradation	0.429	0.778
Glutamate degradation I	0.439	0.778
Nitric oxide synthesis II (nitrite reductase)	0.455	0.778
Serotonin synthesis I	0.455	0.778
Isovaleric acid synthesis II (KADC pathway)	0.480	0.778
Menaquinone synthesis (vitamin K2) II (alternative pathway: futalosine pathway)	0.481	0.778
Dopamine synthesis	0.488	0.778
Acetylcholine synthesis	0.495	0.778

Acetate synthesis II	0.508	0.778
GABA synthesis II	0.536	0.796
Acetate synthesis I	0.579	0.811
S-Adenosylmethionine (SAM) synthesis	0.579	0.811
Nitric oxide degradation I (NO dioxygenase)	0.717	0.957
Histamine synthesis	0.733	0.957
Melatonin synthesis	0.761	0.957
Kynurenine synthesis	0.780	0.957
Glutamate synthesis II	0.782	0.957
Inositol synthesis	0.815	0.957
Acetate synthesis III	0.848	0.957
Nitric oxide synthesis I (NO synthase)	0.848	0.957
ClpB (ATP-dependent chaperone protein)	0.882	0.957
17-beta-Estradiol degradation	0.898	0.957
Propionate degradation I	0.898	0.957
Menaquinone synthesis (vitamin K2) I	0.932	0.972
Glutamate degradation II	0.966	0.986
Nitric oxide degradation II (NO reductase)	1.000	1.000

Supplementary Table S11. Exploratory associations between SCFA-related pathways and clinical or biomarker variables.

Pathway/module	Variable	Spearman rho	p	FDR
Propionate synthesis I	NfL	0.456	**0.013**	0.194
Butyrate synthesis I	EDSS	0.33	0.081	0.417
Propionate synthesis III	GFAP	-0.327	0.084	0.417
Propionate synthesis II	NfL	0.297	0.118	0.443
Butyrate synthesis I	GFAP	0.248	0.193	0.471
Butyrate synthesis I	NfL	-0.23	0.229	0.471
Propionate synthesis III	NfL	-0.21	0.274	0.471
Butyrate synthesis II	NfL	-0.208	0.279	0.471
Butyrate synthesis II	EDSS	0.206	0.283	0.471
Propionate synthesis I	EDSS	0.11	0.569	0.852
Butyrate synthesis II	GFAP	0.091	0.639	0.852
Propionate synthesis II	GFAP	-0.075	0.697	0.852
Propionate synthesis III	EDSS	0.065	0.738	0.852
Propionate synthesis I	GFAP	-0.046	0.811	0.869
Propionate synthesis II	EDSS	0.004	0.984	0.984

Supplementary Table S12. Exploratory associations between tryptophan metabolism pathways and neurological biomarkers.

Pathway/module	Biomarker	Spearman rho	p	FDR
Tryptophan degradation	GFAP	-0.342	0.070	0.418
Tryptophan synthesis	S100B	-0.226	0.239	0.681
Tryptophan degradation	S100B	0.184	0.341	0.681
Tryptophan degradation	NfL	-0.118	0.541	0.812
Tryptophan synthesis	GFAP	-0.039	0.839	0.863
Tryptophan synthesis	NfL	0.034	0.863	0.863

Supplementary Figure S1. Baseline relative abundance of bacteria (V0)

1a. Baseline relative abundance of bacterial phylum

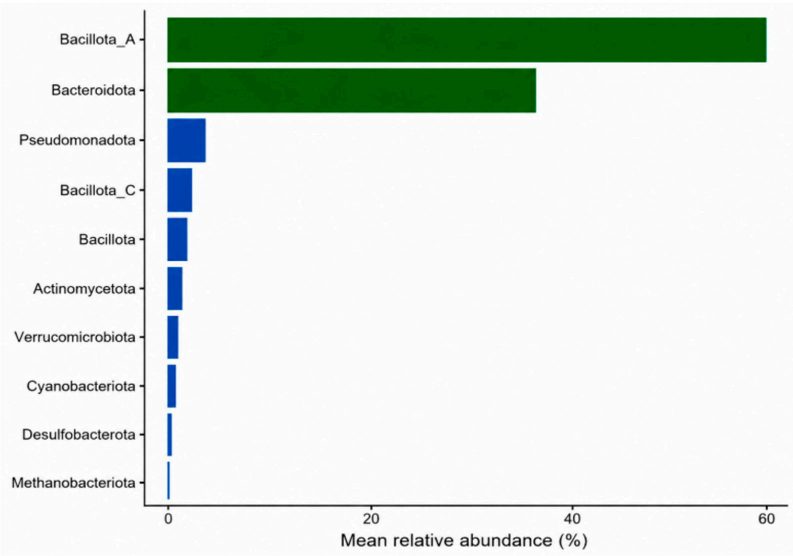

1b. Baseline relative abundance of bacterial genera

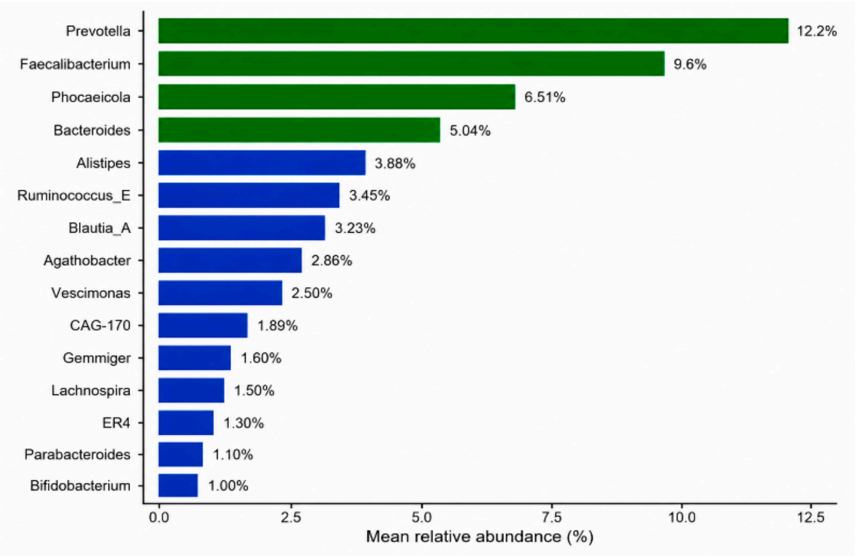

Supplementary Figure S2. Alpha diversity Shannon index for both visits

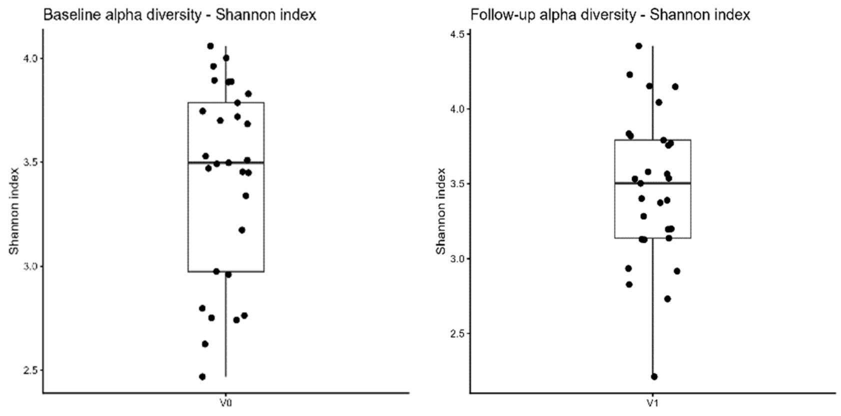

Supplement: Supplementary file 1 [file microorganisms-14-02080-s001.zip › microorganisms-4526264-supplementary.pdf]
